# NMR Meets Tau: Insights into Its Function and Pathology

**DOI:** 10.3390/biom6020028

**Published:** 2016-06-07

**Authors:** Guy Lippens, Isabelle Landrieu, Caroline Smet, Isabelle Huvent, Neha S. Gandhi, Benoît Gigant, Clément Despres, Haoling Qi, Juan Lopez

**Affiliations:** 1 Unité de Glycobiologie Structurale et Fontionnelle, University of Lille (UGSF), Villeneuve d’Ascq CNRS UMR Lille 8576, France; isabelle.landrieu@univ-lille1.fr (I.L.); caroline.smet@univ-lille1.fr (C.S.); isabelle.huvent@univ-lille1.fr (I.H.); clement.despres@ed.univ-lille1.fr (C.D.); haoling.qi160687@gmail.com (H.Q.); juan.lopez@pucp.edu.pe (J.L.); 2 Laboratoire d'Ingénierie des Systèmes Biologiques et des Procédés (LISBP), University of Toulouse, CNRS, INRA, INSA, Toulouse 31400, France; 3 School of Mathematical Sciences, Queensland University of Technology, Brisbane 4001 QLD, Australia; neha.gandhi@qut.edu.au; 4 Institute for Integrative Biology of the Cell (I2BC), CEA, CNRS, Université Paris-Sud, Université Paris-Saclay, Gif-sur-Yvette 91190, France; Benoit.Gigant@i2bc.paris-saclay.fr; 5 Sciences Department-Chemistry, Pontifical Catholic University of Peru (PUCP), Av. Universitaria 1801, Lima 32, Peru

**Keywords:** Tau, NMR spectroscopy, intrinsically disordered protein, tubulin, aggregation, phosphorylation, protein/protein interactions

## Abstract

In this review, we focus on what we have learned from Nuclear Magnetic Resonance (NMR) studies on the neuronal microtubule-associated protein Tau. We consider both the mechanistic details of Tau: the tubulin relationship and its aggregation process. Phosphorylation of Tau is intimately linked to both aspects. NMR spectroscopy has depicted accurate phosphorylation patterns by different kinases, and its non-destructive character has allowed functional assays with the same samples. Finally, we will discuss other post-translational modifications of Tau and its interaction with other cellular factors in relationship to its (dys)function.

## 1. Introduction

The tubulin-associated unit (Tau) is a remarkable protein. First discovered as a protein factor that promotes the assembly of tubulin into microtubules (MTs) [1], it gained further notoriety upon its identification as the principal component of the tangles that characterize neurons of patients with Alzheimer’s disease (AD) [2,3,4,5]. It was additionally one of the first recognized examples of an intrinsically disordered protein (IDP) [6], but how exactly it exerts its function(s) is still a point of debate. Functions other than MT stabilization in the neuronal axons have more recently been described, and concern both synaptic [7] as well as nuclear [8,9] localizations. The possible spreading of (toxic) Tau forms from one neuron to another is another tantalizing aspect that might be at the origin of the spatio-temporal hierarchy of AD [10,11], but a clear molecular definition of the propagating species—monomer [12,13], dimer [14] or oligomers [15,16,17]—is not yet available. Finally, post-translational modifications (PTMs) seem to regulate its function or dysfunction in a most complex manner. Phosphorylation is the most studied PTM, and AD Tau is often described as a hyper-phosphorylated form [18], although it is not clear whether phosphorylation of specific sites is critical for AD, and a stoichiometric association between phosphorylation and AD has not been shown. Equally, its implication in the spreading of Tau is still a subject of debate [19]. Recently, other modifications such as lysine acetylation [20,21] or proline isomerization [22] have also been linked to its dysfunction, but the field lacks mechanistic insights in how these modifications might interfere with its function and/or promote its aggregation. Therefore, despite having been discovered nearly 40 years ago, Tau still mobilizes extensive research efforts.

**Figure 1:**
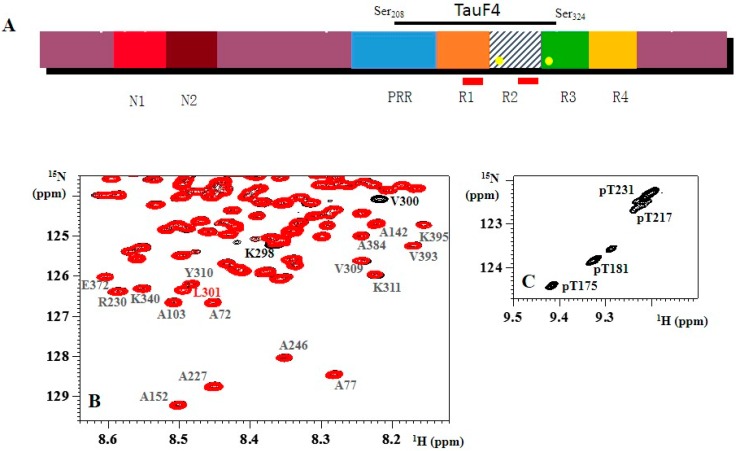
(**A**) Schematic view of the primary sequence of Tau441, the longest isoform of Tau. Different isoforms are characterized by the insertion of 0, one or two N-terminal inserts (N1 and N2), and three (R1-R3-R4) or four (R1-4) repeats (leading to the 3R or 4R forms). The repeats are preceded by a proline-rich region (PRR). The two hexapeptides PHF6 and PHF6* are indicated as red rectangles in R1 and R2, whereas Tau’s two cysteine residues (Cys291 and Cys322) are indicated as yellow circles in R2 and R3. The fragment TauF4 spans part of the PRR, the first two repeats and a small part of R3; (**B**) The ^1^H, ^15^N HSQC spectra of ^15^N-labeled wild-type (black) and ^15^N,^13^C-labeled P301L (red) Tau441 show that only a couple of residues directly adjacent to the mutation show chemical shift differences. Residues Val_309_-Tyr_310_-Lys_311_ of the PHF6 peptide are identical in chemical shift and intensity in both proteins; (**C**) Zoom of the ^1^H, ^15^N spectrum of CDK2 phosphorylated Tau around the resonance of pThr231, showing several peaks for the same phosphorylated residue.

Over the last 10 years, we and others have applied biophysical approaches including high-resolution NMR spectroscopy to study both the functional and pathological aspects of Tau. The assignment of the ^1^H, ^15^N HSQC spectrum has been a long-time technical effort that we started 10 years ago [23,24,25,26,27], and we have been joined in this effort by other research groups [28,29,30] to such a point that the spectrum of the longest isoform (Tau441, Figure 1A) has been completely assigned. In this review, we will not consider the technical progress that has led to this full assignment, but rather focus on what we have learned from these NMR studies, considering both the mechanistic details of the Tau-tubulin relationship and of the aggregation process. Phosphorylation of Tau is intimately linked to both aspects. Combining *in vitro* modification of Tau with recombinant kinases and/or brain extracts and the analytical power of NMR spectroscopy has allowed deciphering the exact modification pattern of the various kinases [31,32,33,34], and the non-destructive character of the analysis implies that the same samples could be further used for functional assays, thereby enabling us to link the PTMs to (dys)function.

### 1.1. NMR Spectroscopy of Isolated Tau

The ^1^H, ^15^N HSQC spectrum of Tau is characterized by a very narrow range of chemical shift values for the amide protons (Figure 1). The nitrogen range is close to normal, reflecting the finding that the nitrogen chemical shift depends more on the nature of the amino acid than on its three-dimensional (3D) environment. Tau is highly degenerated in its amino acid composition, with five amino acids—glycine, serine, lysine, proline and threonine—making up over 50% of its primary structure, but its longest isoform with 441 amino acids contains enough residues to fill the spectral range of 105–125 ppm. Every cross-peak in this spectrum represents one amino acid as a time average over the multiple conformations that it might adopt in the polypeptide. In a protein with a stable fold, this average reflects the exact 3D environment of the amide moiety and will be distinct for every amino acid. In an IDP, however, the average is over the whole conformational space of the amino acid samples, blurring out the environment of any given residue. A consequence is the reduced amide proton chemical shift, which together with the large size of the protein leads to a very crowded spectrum, and it is close to the random coil chemical shift values for the carbon nuclei. The *a priori* acceptation of its IDP nature implies that the carbon chemical shifts become a known parameter, and thereby allow it to return to the ^1^H, ^15^N coordinates and hence identify residues in the spectrum [23,24,25,35]. Later advances including high dimensionality spectra have led to the full assignment of Tau’s spectrum [27,28,30,36]. Resuming what have we learned from this effort, we can note several points. Firstly, beyond a high-tech confirmation of the lack of stable secondary or tertiary structure elements in the isolated protein, the PHF6 (V_306_QIVYK_311_) and PHF6* (V_275_QIINK_280_) hexapeptides previously identified as aggregation nuclei [37,38] have some tendency to sample the β-sheet conformation [39,40]. Although there are examples where a pre-structure is not required or is not found back in the bound conformation [41], the residual β-sheet tendency of the hexapeptides could be important for the mechanism of aggregation. The identification of an essential methyl/π interaction between the Ile308 γCH_3_ methyl and the Tyr310 aromatic ring was interesting, whereby mutational analysis has underscored its importance for the aggregation process [42,43]. Detected at the level of a small peptide, we recently could confirm this interaction in the full-length protein [44].

NMR can be used not only to look at the local secondary structure but also at the preferential global conformations within the ensemble of accessible structures. Spin labeling of Tau via an introduced cysteine with a group carrying an unpaired electron has confirmed a transient folding-back of Tau’s N- and C-termini over the middle of the protein [28,45], as proposed in the “paper-clip” model obtained by FRET measurements on tryptophan mutants of Tau [46]. However, the spectrum of full-length Tau being nearly identical to the sum of the spectra of its fragments argues against any stability of this folded conformation. Moreover, the functional relevance of this fleeting conformation and, even more, the conclusion that it would shield the more hydrophobic microtubule binding regions (MTBRs) from aggregation are far from obvious.

Secondly, the NMR spectrum of wild-type (wt) Tau compared to that of its (pathogenic) mutants strongly suggests that the distinct disease progress associated with those point mutations is not related to a different behavior of the soluble protein. As an example, we show in Figure 1B a superposition of the spectra of wt Tau441 and its P301L mutant, whereby the latter leads to an early form of dementia called FTDP-17 [47,48]. Both spectra were acquired on a single sample, in which ^15^N-labeled wt Tau44 was mixed with ^15^N, ^13^C-labeled TauP301L, and isotope filtering was used to separate the spectra of both proteins [49]. The perfect superposition of both sub-spectra (after correcting for the isotope effect of the ^13^C nucleus on the ^15^N chemical shift) except for the couple of residues in the immediate environment of the mutation, and this for all temperatures between 4 °C and 30 °C, indicates that Tau’s residues sample the same conformational space irrespective of the proline or leucine in position 301.

Thirdly, the NMR spectrum has formed the premise for the study of the phosphorylation of Tau. Phosphorylated serine or threonine residues experience a pronounced downfield shift for their amide proton signals [50], and analysis of the resulting peak intensities allows quantifying the phosphate incorporation at every individual site in a non-destructive manner. Whereas for most phosphorylation patterns that we and others have studied by NMR [32,33,34,51,52,53] the only changes in the spectra involve the residues that are phosphorylated or their direct neighbors, there are some notable exceptions. The first one is Thr_231_, whose phosphorylation induces an upfield shift for the amide correlation of up to 10 downstream residues, due to stabilization of a small α-helix in this region [54,55]. In addition, the resonance of phospho-Thr_231_ splits up in five or more peaks when Tau is phosphorylated on other positions by an activated CDK or Erk kinase (Figure 1C). This implies that the Thr_231_ site “feels” the influence at a long distance from other phosphorylation events, integrating information in a manner that we still do not understand completely. A second example where phosphorylation induces structure is the AT8 epitope, whereby phosphorylation at the Ser_202_ and Thr_205_ positions is “read” by the Arg_209_ and Arg_211_, to form an unusual turn conformation [56]. We will come back to this particular example in detail, but it demonstrates that PTMs can indeed change the structure of Tau in a subtle way.

### 1.2. NMR Spectroscopy of Tau-Tubulin Complexes

Beyond the information gleaned from the spectra of the isolated protein, NMR has been applied to gain information about Tau in its relationship with tubulin. The first experiments combined taxol-stabilized microtubules (MTs) with Tau, and confirmed the presence of the N-terminal projection domain next to a stretch that tightly binds to the mesoscopic MT [57,58]. Signal disappearance characterizes the latter interacting domain, and it could be mapped to part of the proline-rich region (PRR) and the different MTBRs. These findings mainly confirmed what was known from careful affinity measurements with different fragments [59,60,61,62]. The most interesting aspect obviously would be to directly observe the bound conformation—if the latter exists, because the absence of electron density in different cryo-electron microscopy studies of the Tau-MT complex [63,64,65] and the rapid diffusion of Tau on the taxol-stabilized MT surface [66] all suggest that flexibility remains a defining factor, even for MT-bound Tau. However, solution NMR cannot readily access the bound part, as lines are broadened beyond detection by the slow tumbling of the MT. Transfer-NOE studies [67,68] can indirectly “read” the bound conformation by measuring the NOE transfer between protons on the free (NMR-visible) molecule that happened when it was bound (and thus NMR-invisible). One crucial condition is the rapid exchange between bound and free forms [69], which roughly translates in a dissociation constant above the µM range. Using small peptides derived from Tau, Kavadath *et al.* derived the structure of several Tau peptides, and suggested that residues 269–281 and 300–312 would fold in a well-defined structure [70]. However, as recognized by the authors, the NOE contacts might come from different conformations. A second potential problem is that the transfer-NOE method requires a large excess of peptide over tubulin. This might lead to clustering of the peptides on the MT surface, as observed previously for Tau [71], or even to fiber formation on the MT surface [72]. The peptides encompass the aggregation-prone PHF6 and PHF6* sequences [37,38], so the derived structures might reflect the packing in a (pre-) fiber structure at the surface rather than the direct binding to the MT surface. A most promising approach to obtain structural data on the MT-bound Tau conformation(s) would be solid-state NMR (SS NMR). Applied to other MT binding proteins such as Cap-Gly [73], this approach has shown its capacity to overcome the size limitations imposed by solution NMR, and should be applicable to the Tau-MT complex.

**Figure 2:**
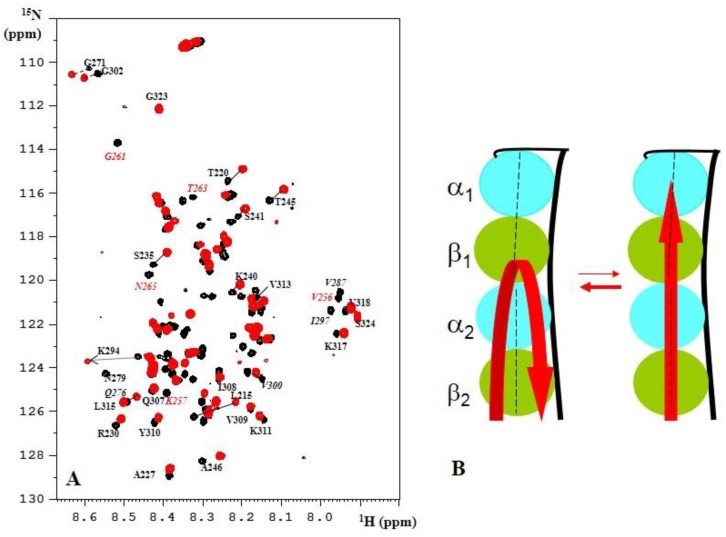
(**A**) The ^1^H, ^15^N TROSY spectrum of TauF4 in its complex with T_2_R. Residues in the R1 repeat whose resonances are absent in the TauF4-T_2_R spectrum are labeled in red; (**B**) Model of the swing movement of TauF4 on the curved T_2_R surface [76].

Several reports have hinted at the possibility of different binding modes for Tau when it binds to the taxol-stabilized MT or when it is itself the polymerizing agent [74,75]. Defining the latter conformation is, however, a formidable challenge. A different strategy is to approach the mechanism of Tau-promoted MT assembly by considering its binding to the first tubulin heterodimer(s) before assembly has happened. Adopting this approach, we used assembly-incompetent tubulin constructs to gain mechanistic insights into how Tau might regulate tubulin dynamics [76]. We started with the T_2_R complex, wherein two tubulin heterodimers are sequestered in a curved conformation by the stathmin-like domain of Rb3 (Rb3_SLD_) [77,78]. Clearly, such constructs cannot answer questions about the role of Tau in lateral contacts, as suggested by the different Tau isoforms regulating the number of protofilaments in the MT [79]. Initial attempts to solve the crystal structure of this T_2_R-Tau complex proved quite frustrating: all protein partners were present in the crystal, but only the tubulin heterodimers and Rb3_SLD_ were visible in the electron density maps, whereas no signal could be attributed to Tau. To limit the complexity of an NMR study on the same complex, Tau was first partially digested and its T_2_R binding fragments isolated by gel filtration. Connecting two of these short binding fragments led to the TauF4 (Tau 208–324) construct (Figure 1A) which not only binds more tightly than full-length Tau to the MT surface but equally is very efficient in MT assembly [80]. NMR studies of a labelled TauF4 fragment on T_2_R firstly gave indications of the mobility of the fragment on the surface. Notably the PRR region and the C-terminal PHF6 peptide showed narrow lines (Figure 2), allowing for their unambiguous assignment in the complex [76]. Carbon chemical shifts of residues in these fragments were identical to those of the isolated TauF4 fragment, arguing against any stable secondary structure for them at the tubulin surface. Residues in the first two repeats did not show signals, suggesting they are at least partially immobilized on the surface. Surprisingly, using a complex comprising one tubulin heterodimer sequestered by a modified stathmin-like domain protein as a binding target [81], some signals of residues centered around Ser_262_ became visible while, simultaneously, resonances of the PHF6 residues became severely broadened. This suggested a model wherein TauF4 localizes as a U-turn on the first α/β tubulin heterodimer, with the I_260_GSTEN_265_ peptide protruding from the surface of the α subunit. After binding to a first tubulin heterodimer, TauF4 can recruit a second tubulin heterodimer with its protruding peptide. Importantly, phosphorylation of Ser_262_ by the MARK kinase leads to complete loss of Tau’s capacity to assemble MTs [82]. The binding of a second tubulin heterodimer transmits to the C-terminus of the Tau fragment, setting free the PHF6 residues that thereby can swing to the incoming tubulin and possibly induce its straight conformation (Figure 2). In the T_2_R complex, however, the curved conformation is stabilized by the Rb3_SLD_ helix, so TauF4 would oscillate between both the U-turn and extended conformation. SS NMR with TauF4 fragments spin-labeled at different positions might confirm whether TauF4 does adopt an extended conformation on the straight MT surface.

In another recent study, the competition of Tau with the tubulin-targeting small compound vinblastine led the authors to conclude that Tau would bind at the interface between the αβ tubulin heterodimers [83]. However, this particular vinblastine site identified in the T_2_R complex does not exist anymore in the straight microtubule, as it collapses when tubulin goes from its curved to straight form [84]. Vinblastine binds also to a number of weaker affinity sites along the surface of the microtubule [85], and this might explain the competition. The localization of Tau on the MT surface by NMR spectroscopy will be particularly arduous, as most studies rely on unlabeled tubulin purified from different animal sources. Recently, several sources of recombinant tubulin have been reported [86,87,88]. Stable isotope labeling of the tubulin subunits hence might become feasible, opening up a way to map in further detail the Tau-tubulin interface.

### 1.3. Aggregation of Tau

The aggregation of Tau during the development of AD has gained further interest as a pharmaceutical target over the last 10 years, especially as clinical tests aimed at the aggregation of the Aβ amyloid peptide have regretfully not led to improved cognitive functions of patients. Fundamental insights in the process have built up ever since the discovery of Tau as the main component of the intraneuronal tangles [2,3,5], but a large number of open questions remain. Initial efforts were aimed at characterizing the brain-derived fibers that show up as paired helical filaments (PHFs) or straight filaments (SFs). This led to the recognition of the hyper-phosphorylated state of Tau in the fibers [18] as well as to the identification of its MTBRs as the core of the fibers [89]. The reproduction of the process in a test tube was initially unsuccessful with the full-length Tau, and proved only feasible after the long incubation of selected fragments of Tau encompassing its MTBR as the core domain of natural fibers [90]. The necessity of an intermolecular disulfide bridge [91] and the identification of the aforementioned PHF6 and PHF6* peptides as nucleation sites of the process [37,38] were two important results. A solid phase assay based on the adsorption of a Tau fragment on a PVC microtiter plate and the subsequent binding of Tau notably reproduced the protease resistance of the PHF core, and allowed the identification of several small molecules as potential inhibitors of the Tau-Tau interaction [92]. Aggregation starts from the absorbed Tau at the negatively charged PVC surface of these microwells, and is closely related to the aggregation that occurs at the surface of arachidonic acid micelles [93]. Both assays seemingly do not require the cysteine oxidation of Tau to promote Tau fiber formation [94]. When heparin and other poly-anions were identified as aggregation-promoting factors even of full-length Tau [95,96,97], and Thioflavin fluorescence as a convenient way to monitor the amyloid component of fibrils of Tau [98], the door was opened for massive screening for potential inhibitors of Tau aggregation [99,100,101]. The literature abounds with small molecules that inhibit the *in vitro* process of Tau aggregation. Surprisingly, however, only methylene blue, identified in the solid phase Tau binding assay years before the high-throughput screens gained in popularity [92], has currently entered the clinic as a potential disease-modifying agent [102]. Whether this compound acts through oxidation of the cysteine residues of Tau [103,104] or some other mechanism [105] remains an open but important question.

Structural characterization of Tau fibers started by assigning those residues that still maintain a signal in the NMR spectrum of macroscopic fibers, indicating their residual mobility [106,107,108]. Other techniques combining mass spectroscopy of fibers after limited proteolysis [109] or hydrogen/deuterium exchange mass spectrometry [110] equally confirmed the overlap of the core of the fibers with the MTBR [89], but did not tell more about the core structure itself.

**Figure 3:**
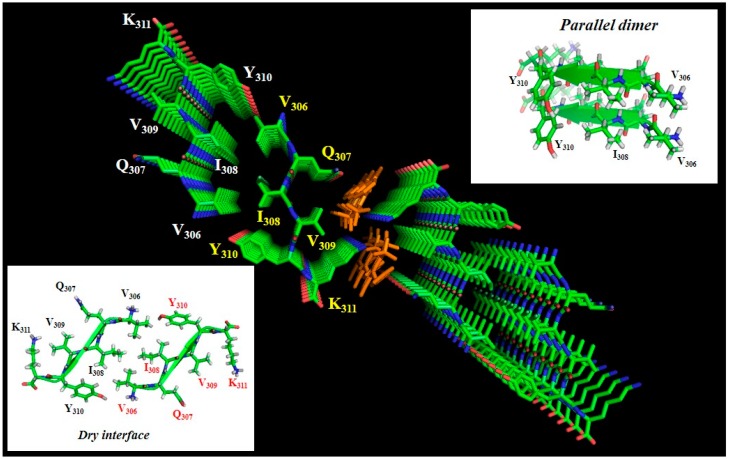
Crystal structure of the PHF6 peptide in complex with orange G (PDB code 3OVL), seen along the fiber axis from the top. Inserts show the antiparallel dry interface between opposing peptides, or the parallel stacking between adjacent peptides in the same plane. Orange G has two sulfate groups separated by 5 Å, and thereby compensates the lysine side-chain positive charges of two adjacent peptides [120].

However, akin to the situation of the limited data on the exact Tau-tubulin interface, detailed conformational information on Tau fibers remains scarce, especially when we compare it with the available atomic-level information on other amyloid proteins, such as the Aβ peptide or the yeast prion proteins [111,112]. Polymorphism of the fibers [113] has been extensively characterized for these latter proteins [114], and physiologically relevant structures have been produced by seeding experiments starting from brain-derived Aβ plaques and recombinant Aβ peptides [115]. Similar seeding experiments for Tau starting from AD brain-derived extracts have been described [116,117], but no structural studies at the per atom level have been reported for these templated fibers. Reasons for the limited data on the molecular organization of Tau fibers are the size of the protein, the necessity to use an exogenous poly-anion to obtain large quantities of fibers, the different isoforms of Tau that do not necessarily adopt the same organization, and evidently the ever-present doubt of whether the synthetic fibers one can make on a bench represent the intraneuronal fibers present in AD patients. Insight into the molecular organization of the fiber core presently comes mainly from three sources. First, X-ray crystallography [118] on the small hexapeptides that nucleate the aggregation points to a parallel stacking of these peptides inside a first sheet that is complemented by a second sheet in which the peptides are arranged antiparallel to their counterparts in the first sheet, thereby forming a dry interface (Figure 3). Although not directly a structure of the fibers, these crystal structures provide a molecular model that has been used to develop potential breakers of this pattern in full-length Tau fibers [119].

**Figure 4:**
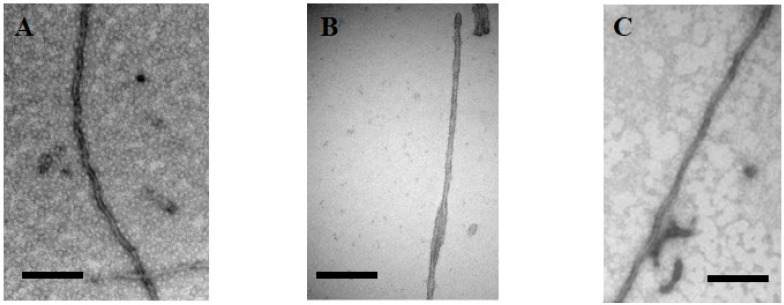
Electron microscopy of (**A**) heparin-induced synthetic fibers of Tau441 show the morphology of paired helical filaments, whereas (**B**) those of a TauF4 fragment devoid of cysteine residues show a morphology of two flat ribbons twisted around one another [126]. This morphology is found in the *ex vivo* 3R fibers characterizing Pick’s disease [127]; (**C)** TauP301L after phosphorylation by Erk2 forms fibers without heparin. Scale bar = 100 nm.

Secondly, solid-state NMR on fibers of a short K19 fragment (corresponding to the MTBR of the 3R fetal isoform) has confirmed the presence of three major β-sheets, with one encompassing the aforementioned PHF6 peptide [121]. Intriguingly, the intermolecular disulfide bridge that can be formed by the single Cys322 in this K19 fragment shows structural heterogeneity in the SS NMR spectra [122]. Finally, electron paramagnetic resonance (EPR) spectroscopy on Tau samples labeled by a single spin label or two spin labels in the monomer has been used to deduce the mobility of residues in the fibers (by continuous-wave EPR) and distances between two spin labels within a single protein (by double electron-electron resonance or DEER) [123], and hence provides insights into their spatial arrangement in the fibril cross-section. Individual Tau proteins would thereby form single-molecule layers along the fiber axis, which perfectly stack on top of each other by in-register, parallel alignment of β-strands [124]. Next to the finite size of the spin label, one caveat for the EPR work in relationship to Tau fibrils is the need to introduce a single cysteine in the monomer for labeling purposes. This implies removing the native cysteines at positions 291 and 322, whereas these residues play an important role in fiber formation and even its final organization. As a simple example, we investigated this aspect in the context of TauF4, whose C-terminal truncation at Ser324 allows for efficient fiber formation [125]. The fibers of the fragment without the cysteines, rather than the rope-like twisted fibers that characterize AD, display the typical sheet morphology found in Pick’s disease, although this disease is characterized by 3R Tau fibers (Figure 4), further underscoring the possible polymorphism of Tau fibers.

As the molecular mechanism(s) that transform the highly soluble IDP that is Tau into an aggregation-prone species are not known, the majority of structural studies use poly-anions such as heparin to accelerate the *in vitro* aggregation process [95,96]. Although heparin might play a role in the transmission of certain Tau species from neuron to neuron [128] and might even be internalized from the cell surface in certain conditions [129], there is no direct evidence that it would be the causative agent for intraneuronal aggregation. The role of heparin is also not well defined in the aggregation process: as a highly negatively charged molecule, does it merely bind several Tau molecules, thereby increasing their local concentration above a critical concentration [130]? Is there some specific sulfation pattern required to bind to defined lysine residues of the MTBR and induce a conformational transition [129,131]? Negatively charged orange G binds specifically to lysine side-chains of adjacent sheets in the peptide crystal (Figure 3), suggesting that a negative charge at 5 Å separation would be essential for charge compensation [120]. Heparin indeed does enter the core of the fibers, suggesting that it plays a role in charge neutralization of the lysine ladders that would originate from parallel in-register stacking of the PHF6 and/or PHF6* peptides [132].

## 2. Phosphorylation and Tau

With its 45 Ser, 35 Thr and 5 Tyr, the longest isoform of Tau (Tau441) can in principle be phosphorylated over nearly one-fifth of its length. Although such levels of phosphate incorporation have never been observed, phosphorylation at multiple sites does seem a defining factor for both its physiological and pathological aspects [133,134,135,136]. However, the translation of a given phosphorylation pattern into functional consequences is subtle, as can be appreciated from the following examples.

Ser_262_, in the first MTBR repeat, can be efficiently phosphorylated by the MARK kinase, whereby the acronym stands for “Microtubule Affinity Regulating Kinase” [82]. Indeed, in a co-sedimentation assay whereby MARK-phosphorylated Tau is mixed with taxol-stabilized microtubules, more phospho-Tau remains in solution after spinning down the MTs when compared with unphosphorylated Tau [137]. This was interpreted in terms of a reduced affinity imposed by the phosphorylation of this residue, but Tau can form fibers at the surface of MTs that equally will sediment in the assay [72]. Phosphorylation at the Ser_262_ position can impede fiber formation [137], and might as such reduce the amount of sedimented material. Phosphorylation of Ser_262_ unambiguously prevents Tau’s capacity to assemble microtubules, in agreement with the mechanistic insights we described above, but whether it really detaches Tau from the MT surface asks for further investigations.

Ser_214_, in the PRR, is a residue that is readily phosphorylated by the PKA kinase [51]. Its phosphorylation lowers the affinity of Tau for the MT surface [57]. The regulatory role of the PRR for the Tau/MT interaction was previously deduced from affinity measurements of different fragments [61,62], but how phosphorylation regulates this is still not clear. In our study of CDK2/CycA3-phosphorylated Tau, we found, at best, a modest drop in affinity despite four residues in the PRR (Ser_202_, Thr_205_, Thr_231_ and Ser_235_) being modified [32]. Affinity regulation in the Tau/MT complex hence does not depend on the mere level of phosphorylation, as was proposed for other complexes [138]. The large chemical shift perturbation for the amide proton of Leu_215_ within the TauF4 fragment in its complex with T_2_R [76] rather points to a more specific effect of some of these phosphorylation events, although the specific interaction spot(s) on the tubulin surface remain to be mapped. Phosphorylation of Ser_214_ also interferes with the heparin-induced aggregation of Tau [137], although it is outside the core region as determined by different biochemical and/or biophysical methods. Clearly, the PRR has a regulatory role in Tau’s (dys)function that can be modulated by specific phosphorylation events in an as yet poorly understood manner.

Clinical post-mortem staging of AD currently relies on immunochemical detection of phosphorylated Tau by the AT8 antibody. Earlier work identified the epitope of this antibody as a peptide centered on the pSer_202_/pThr_205_ phosphorylation sites [139,140,141]. Most recently, the crystal structure of the AT8 Fab with several phospho-peptides confirmed this motif as a possible epitope, but showed even tighter binding to a triply phosphorylated pSer_202_/pThr_205_/pSer_208_ peptide [142]. This most probably solves the long-standing question of the developmental regulation of the AT8 antibody immunoreactivity. It was recognized early on as reactive against fetal Tau as well as AD Tau, but with a vanishing immunoreactivity for adult healthy Tau [143]. Our guess would be that the fetal form concerns only the pS_202_/pT_205_ phosphorylation, whereas the additional phosphorylation at Ser_208_, one of the few sites detected uniquely in AD brains [135], would be specific to AD Tau.

**Figure 5:**
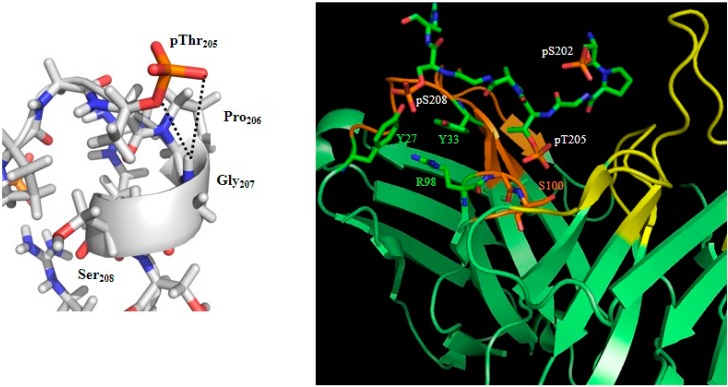
Structure of the AT8 motif in solution [56] or in complex with the AT8 Fab fragment ([142]; PDB code 5E2W). The helical turn stabilized by the interaction between the Gly_207_ HN and the phosphate group of pThr_205_ (left) is not maintained in its complex with the AT8 antibody (right). The phosphate group of pSer_208_ makes additional interactions with residues of the antibody. The AT8 Fab is in green, with the variable loops of the light and heavy chain in yellow, respectively, orange.

Accurate epitope determination of the antibodies used in a research or clinical setting is of the utmost importance, and NMR can play an important role in this [56,144,145]. Recently, we determined that pS_202_/pT_205_ phosphorylation in Tau induces a helical turn conformation, whereby the phosphate of Thr_205_ hydrogen bonds to the amide proton of Gly_207_ (Figure 5). This conformation, readily detected through the downfield NMR shift to 9.4 ppm of the amide proton of Gly_207_, is however not completely stable, especially when we compare with the two identical turns induced by the phosphorylation of Thr_37_ and Thr_46_ in the intrinsically disordered 4E-BP2 [146]. This major neural isoform of the family of three mammalian proteins that bind eIF4E and suppress cap-dependent translation initiation forms a triple-stranded β-sheet after phosphorylation, and the amide protons of the two Gly residues in the identical Thr-Pro-Gly motifs shift to 11 ppm after phosphorylation of the Thr side-chains [146]. The AT8 Fab fragment binds the pS_202_/pT_205_ peptide not as a helical turn but rather in a polyproline II helical conformation, whereby the pSer_202_, now hydrogen, bonds with the amide of Gly_204_, thereby providing an N-terminal cap for the bound conformation [142]. The pThr_205_ side-chain in the AT8-bound conformation interacts with the Gly_99_-Ser_100_ of the antibody (Figure 5) rather than with its own Gly_207_-Ser_208_, as is the case in the free form. The side-chain of pSer_208_ is coordinated by the side-chains of Tyr_27_, Tyr_33_ and Arg_98_ of the antibody, and thereby contributes significantly to the higher interaction strength.

Is phosphorylation a (or the) trigger for the *in vivo* aggregation of Tau? This hypothesis rests mainly on the results of the Iqbal group [147,148,149], who particularly showed that after *in vitro* phosphorylation by a rat brain extract, the resulting sample contains nine to 12 phosphates per molecule and aggregates into PHF-like structures after incubation at 37 °C without the addition of any poly-anions [149]. Amazingly, although this paper has been cited over 400 times, we are not aware of independent confirmations of this important result. Exploiting the extensive phosphorylation of Tau when expressed in Sf9 insect cells, Tepper *et al.* concluded that the highly phosphorylated protein readily forms oligomers, but fibrils where only rarely observed [150]. Recently, we determined that the Erk2 stress-related kinase can phosphorylate Tau on many more sites than any other kinase we studied before, at a level close to that of a rat brain extract [34]. We did obtain fibers with a morphology similar to the PHF extracted from AD brain, both with samples phosphorylated by the isolated Erk2 kinase or by the rat brain extract. The amount of fibers, however, again was low. When using the P301L mutant of Tau in combination with Erk2 phosphorylation of Tau, fibers were observed by electron microscopy only in the phosphorylated sample, with a similar PHF morphology as observed for the heparin-induced fibers of unmodified Tau (Figure 4), but precise quantification was still not possible [151]. These combined results suggest that phosphorylation indeed can trigger aggregation, but further work is required to elucidate the complex relationship between both phenomena.

## 3. Other Protein Interactions and PTMs of Tau

Whereas Tau’s axonal role in microtubule assembly and in PHF formation together with the regulation of both aspects by its phosphorylation have dominated the literature over the last 30 years, many other functional and pathological interactions have recently been described. Bin1, one of the first genetic factors linked to Tau pathology [152] that came out of a genome-wide association study of AD patients [153], was shown to interact physically with Tau after phosphorylation of the latter in its PRR region [154]. NMR studies have validated the phosphorylated residues pSer214 and pSer324 in Tau as the two primary sites for the binding of 14-3-3σ [155], a protein that has been identified in the neurofibrillary tangles. Detailed structural studies have then provided the basis of the development of small molecules that can inhibit this protein/protein interaction [156]. The interaction of FKBP52, an immunophilin that can promote Tau aggregation [157,158], was mapped in detail [159]. Heat shock proteins are involved in Tau degradation, but the interaction with different members can determine the final outcome: interaction with the constitutive Hsc70 chaperone slows Tau clearance, whereas the stress-controlled Hsp72 tends to accelerate Tau degradation [160]. The interaction of both chaperones on Tau was mapped to the PHF6/6* peptides, with nevertheless a more pronounced interaction for the Hsp72 stress-induced chaperone [160]. The Hsc70 E165S mutation locks Hsc70 into an ADP-bound conformation, thereby preventing the dissociation of DnaJ and abrogating the interaction with other Hsp70 family members. This promotes the recruitment of Hsp90 to the complex to facilitate Tau degradation [161]. Hsp90 interacts with Tau via an extended surface that involves the PRR region of Tau and its four MTBRs [162]. Its interaction via multiple hydrophobic patches is akin to that with late folding intermediates. Recently, insight has been gained of other PTMs that also could play a role in both the physiological and pathological aspects of Tau. NMR has given some insight into the precise definition and/or possible role of Tau *O*-GlcNAcylation [163] and acetylation [164], but its lesser sensitivity and need for stable isotope labeling render it less attractive as an analytical tool than mass spectrometry. The same cannot be said of the proline cis/trans isomerization, a non-covalent PTM that is purely conformational, and hence leaves no trace in the mass spectrum. Conformation-specific antibodies have led to the notion that Alzheimer’s disease together with other Tauopathies such as traumatic brain injury would be characterized by a specific phospho-Thr_231_-Pro_232_ *cis* bond, and hence classify under the general disease of “cistauosis” [22,165]. Exploiting the sensitivity of NMR chemical shifts to local conformation, we recently showed that this specific prolyl bond in phosphorylated Tau is majorly in the *trans* conformation [166], in agreement with the avian antibody-bound X-ray structure of a Thr_231_-centered phosphorylated Tau peptide [167]. There is, however, an intriguing relationship between Tau and different prolyl cis/trans isomerases. FKBP52 was found to promote the oligomer formation of certain Tau forms [158,168], but detailed NMR analysis of the conformation of the involved prolines refuted that this would be through its prolyl isomerase activity [159]. Pin1, an essential prolyl cis/trans isomerase involved in cell cycle regulation, was shown to regulate the functional [169] and pathological [170] aspects of phosphorylated Tau. Whether this is a direct effect of its prolyl cis/trans isomerase activity or an indirect effect via its regulation of Tau’s (hyper)phosphorylation [171] is not yet clear.

## 4. Conclusions

Because Tau is an IDP, structural methods such as X-ray crystallography and/or cryo-electron microscopy have had limited success in unraveling its functional and pathological aspects. NMR spectroscopy has gone a long way, and now that the spectrum assignment problem is solved, the field can further advance towards questions that directly interest the neurobiology and pharmacology community. Multiple open questions remain: the structural features of Tau in the tau:tubulin co-polymer, the molecular definition of toxicity, the mechanism of action of small molecules that interfere with the Tau oligomer or fiber formation, and how do phosphorylation and other PTMs regulate these aspects, *etc.* We hope that the technique will contribute productively to the goal of understanding, at the molecular level, the regulation of Tau’s (dys) function, and ultimately help to find disease-modifying agents for Alzheimer’s disease and related Tauopathies.
